# Characterization and transcriptomic analysis of a native fungal pathogen against the rice pest *Nilaparvata lugens*

**DOI:** 10.3389/fmicb.2023.1162113

**Published:** 2023-05-18

**Authors:** Zheng-Liang Wang, Yan-Dan Wang, Yi-Qing Cheng, Zi-Hong Ye, Guang-Fu Liu, Xiao-Ping Yu

**Affiliations:** Zhejiang Provincial Key Laboratory of Biometrology and Inspection and Quarantine, College of Life Sciences, China Jiliang University, Hangzhou, Zhejiang, China

**Keywords:** brown planthopper, fungal pathogen, *Aspergillus fumigatus*, transcriptomic sequencing, biocontrol

## Abstract

The brown planthopper (BPH), *Nilaparvata lugens*, is one of the most destructive pests of rice. Given the threats posed by insecticide resistance to its control, eco-friendly strategies based on microbial pathogens emerged as a promising biocontrol alternative. In the present study, we isolated a native fungal pathogen against BPH from infected BPH cadavers and preliminarily identified as a strain of *Aspergillus fumigatus* based on morphological and molecular methods. Laboratory bioassay revealed that this fungal strain was highly virulent to BPH both at nymphal and adult stages, with the median lethal times (LT_50_) of 7.5 and 5.8 days under high conidial concentration of 1 × 10^9^ conidia mL^–1^. A genome-wide view of gene expressions in BPH against fungal attack was analyzed by transcriptomic sequencing and consequently a large number of differentially expressed genes that mainly involved in host immune defense and cell detoxification were found. RNAi-mediated knockdown of an upregulated gene encoding a serine protease (*NlSPN*) could cause a significant decrease in BPH survival. Combination of dsRNA injection and fungal infection showed an additive effect on BPH mortality, which provided clues to develop new pest management strategies against BPH.

## Introduction

The brown planthopper (BPH), the common name for *Nilaparvata lugens* Stål (Hemiptera: Delphacidae), is considered as one of the most destructive pests of rice throughout Eastern and Southeastern Asia. Both nymphs and adults of BPH can pierce and suck the phloem sap, thus causing serious wilt or even complete death of the whole rice plants. Additionally, BPH can transmit plant-pathogenic viruses by a persistent manner, such as the rice ragged stunt virus (RRSV) and the rice grassy stunt virus (RGSV), which further drastically decrease rice yield (Cheng et al., 2013). Currently, application of conventional chemical insecticides is the primary control strategy for BPH. However, the long-term use of synthetic chemicals has generated many negative side-effects, such as environmental pollution and insecticide resistance (Jena and Kim, 2010; Liao et al., 2021). Therefore, it is imperative to search non-insecticidal strategies for BPH control.

A safe and efficient alternative strategy to pest control is the use of insect-pathogenic microorganisms (Lacey et al., 2015). Unlike entomopathogenic bacteria and viruses that invade insects through their oral cavity and/or intestinal tract, fungal pathogens are able to infect their hosts by directly penetrating through the cuticle under natural conditions and hence are considered the only group of insect pathogens that have great potential to control sap-sucking pests (Hussain et al., 2014). Nowadays, accumulating studies have shown that entomopathogenic fungi, in particular two representative genera of *Beauveria* and *Metarhizium*, are effective against different hemipteran pests, including aphids, whiteflies, leafhoppers and scales (Sain et al., 2021; Souza et al., 2021; Xu et al., 2021; Mantzoukas et al., 2022). However, fungal candidates with high efficacy for controlling of rice planthopper are still scarce, although some fungal species have been reported to act against BPH (Li et al., 2014; Wang Z. L. et al., 2021; Zhao et al., 2021). Virulence is the most important indicator for screening high-virulent fungal strains. Generally, native fungal strains that isolated from naturally infected hosts show more virulence than non-native strains (Pirali-Kheirabadi et al., 2007). Hence, there is still a need to find new resource of entomopathogenic fungi that can be used to BPH biocontrol, especially the natural occurrence of fungal strains on BPH.

Entomopathogenic fungi widely exist in nature and generally infect their insect hosts by three concerted stages. Fungal conidia firstly attach to the insect cuticle, then germinate and penetrate the exoskeleton, and finally proliferate in insect haemocoel and kill the hosts (Wang and Wang, 2017). During this process, insect can initiate their innate immune system to defend against fungal infection (Butt et al., 2016; Lu and St Leger, 2016). Obviously, the outcome of the competitive interaction between the specific fungus and its host insect is the major determinant of the fungal pesticidal efficiency. Therefore, a thorough understanding of molecular mechanism of fungus-insect interaction could improve the pest control potential of fungal insect pathogens. Recently, transcriptional response of BPH upon infection with *Metarhizium anisopliae* at different post-infection time points were examined by RNA sequencing, which greatly deepened the understanding of the molecular basis of BPH in response to fungal challenge (Peng et al., 2020; Wang Z. L. et al., 2021). However, the modes of interaction between insect and fungal pathogen are highly complex and specific, the pathogenic strategies used by different entomopathogenic fungal strains to infect the same insect host were not conserved (Wang Y. L. et al., 2021). Hence, the interaction mechanism between each fungus-host pair should be separately investigated for developing an efficient pest control method.

The aim of this study was to isolate and identify a new native fungal pathogen from the infected BPH cadavers collected in paddy field, as well as to evaluate its biocontrol potential against BPH both at the nymphal and adult stages. Our study also attempted to determine the transcriptomic response of BPH to the fungal infection for different periods at a genome-wide level in order to obtain novel insights into the molecular basis for host-pathogen interactions.

## Materials and methods

### Insect rearing

The laboratory BPH population originally established from a paddy field collection was maintained on the rice variety TN1 (Taichung Native 1) under 27 ± 1°C, 70 ± 10% relative humidity and a 14:10 h light/dark photoperiod in the insectary. TN1 rice was growth under the same conditions of BPH-rearing. Third instar nymphs and newly emerged (24–48 h after emergence) adults of BPH were collection for bioassays.

### Fungal isolation and identification

The infected cadavers of BPH were collected from a paddy field in Yuyao, Zhejiang province of China (E121°33, N29°99) and then brought to laboratory for fungal pathogen isolation. In brief, BPH cadavers were suspended in distilled water with 0.02% Tween 80 and shaken vigorously for 3 min. The suspension was diluted 10-fold and 100 μL diluent was spread on potato dextrose agar (PDA) medium. A pure fungal colony was isolated by single conidia purification at 28°C and maintained on PDA at 4°C. The isolated fungal pathogen was initially identified based on morphological characteristics (color, texture of conidia and mycelium) observed under a microscope.

For molecular identification, the fungal culture was grown for 3 days at 28°C on a PDA plate with a cellophane film overlaid on the surface. The mycelia were scraped from the cellophane and homogenized in liquid nitrogen with a mortar. Total fungal DNA was extracted using the Fungi Genomic DNA Extraction Kit (Solarbio, Beijing, China) following to the manufactures protocol. The internal transcribed spacer (ITS) containing the region encoding the ITS1, 5.8S rDNA and ITS2 was amplified by PCR with the universal primers ITS1 (5′-TCCGTAGGTGAACCTGCGG-3′) and ITS4 (5′-TCCTCCGCTTATTGATATGC-3′) (Korabecná et al., 2003). The PCR reaction was performed in a total volume of 50 μL, containing 2 μL template DNA (about 50 ng), 5 μL 10 × PCR buffer, 1 μL dNTPs (10 mM), 1 μL each primer (10 μM), 0.5 μL Taq DNA Polymerase (5 U/μL), and 39.5 μL ddH_2_O. The PCR reaction condition was as follows: an initial denaturation for 3 min at 94°C, followed by 35 cycles of [94°C for 30 s, 55°C for 45 s, 72°C for 30 s], and a final extension step for 5 min at 72°C. The amplified products were checked by agarose gel electrophoresis, cloned into PMD19-T vector (TaKaRa, Japan) and then subjected to pair end sequencing at Sangon Inc (Shanghai, China). The acquired sequence was compared with those of other fungal ITS sequences in the NCBI nucleotide database *via* BLASTn.^1^ The MEGA X^2^ was applied to construct a phylogenetic tree using neighbor-joining (NJ) method with a bootstrap test of 1,000 replicates (Kumar et al., 2018).

### Fungal virulence evaluation

Aerial conidia from a 7-day growth fungal culture on PDA plates were harvested by washing with 2 ml distilled water with 0.02% Tween 80 and adjusted to a final concentration of 1 × 10^9^ conidia mL^–1^. Third-instar nymphs and 24 h newly emerged adults of BPH were separately prepared for fungal virulence evaluation following a protocol published previously with minor modifications (Jin et al., 2011). Briefly, batches of 30–40 nymphs or adults feeding on 5–10 cm high rice seedlings in uncaged cups were sprayed with an equal-volume (1 ml) of conidial suspension (fungal treatment) or 0.02% Tween 80 (control treatment) using a handheld micro sprayer. After spray treatments, all the cups were covered with sterile gauze and placed into a growth chamber at 25°C and a 14:10 h light/dark photoperiod. The survival of BPH nymphs or adults was recorded daily for 10 days. Three replicates were performed for each treatment.

### Transcriptomic sequencing and analysis

For the preparation of samples for transcriptomic sequencing, samples of 100 surviving adults were collected from both the fungal treatment and the control treatment at 1, 2, 3 and 4 days post-infection time points, respectively. At each time point, sampling was conducted in three biological replicates. All the samples were immediately frozen in liquid nitrogen and separately homogenized with a mortar and pestle. Total RNA of each sample was extracted using TRIzol^®^ Reagent (Invitrogen, Carlsbad, CA, USA) and treated with DNase I (New England Biolabs, UK) to remove genomic DNA contamination. RNA concentration was measured using a NanoDrop 2000 spectrophotometer (Thermo Scientific, Wilmington, DE, USA) and RNA integrity was verified by RNase-free agarose gel electrophoresis. Then high-quality RNA from each sample was sent for RNA sequencing on an Illumina MiSeq platform (San Diego, CA, USA) at Lianchuan Biotech Co., Ltd., (Hangzhou, Zhejiang, China) according to manufacturers’ instructions. Briefly, mRNA was purified from total RNA using oligo(dT) magnetic beads. After purification, total mRNA was fragmented into small pieces and then reversely transcribed with random hexamer primers for the first cDNA strand synthesis. The second cDNA strand was synthesized using DNA polymerase I and RNaseH. The cDNA fragments were purified and washed for end reparation and adding poly(A), followed by ligation with sequencing adapters. The cDNA fragments were then purified and enriched by PCR to construct the final cDNA library for high-throughput sequencing.

Clean tags were obtained after removing adaptor sequences, poly(N) tail and low quality sequences from the raw data, followed by mapping to the reference genome databases of *N. lugens* using HISAT2 (v2.0.4) with an allowance of up to one-base mismatches (Kim et al., 2015). Gene abundances were quantified using the FPKM (Fragments per kilobase of transcript sequence per millions base pairs sequenced) method (Trapnell et al., 2010). DESeq R package (1.10.0) was used to determine differentially expressed genes (DEGs) between two treatment conditions (fungal treatment and control treatment) based on the standard of | log_2_ (Fold change)| > 1.0 and the adjusted *P* < 0.05 (Wang et al., 2010). All identified DEGs were then subjected to gene ontology (GO) analysis and kyoto encyclopedia of genes and genomes (KEGG) pathway enrichment analysis at the threshold of corrected *P* < 0.05. The raw data have been submitted to the NCBI sequence read archive (SRA) database under the accession number PRJNA934145.

### Quantitative RT-PCR (qRT-PCR) analysis

To validate the DEGs in the libraries, ten DEGs were randomly selected for quantitative RT-PCR (qRT-PCR) confirmation. The methods for sample preparation and total RNA extraction were the same as those for transcriptomic sequencing. Every 2 μg RNA was reversely transcribed with PrimeScript™ RT kit (Takara, Japan) according to manufacturers’ instructions. The resulting cDNA templates were used for qRT-PCR with SYBR^®^ Premix Ex Taq™ II kit (Takara, Japan). The *18S rRNA* gene (*Nl18S*) was used as the internal standard. The specific primers of genes that were used for qRT-PCR were listed in Supplementary Table 1. All qRT-PCR reactions were performed using an ABI Prism 7500 system (Thermo Fisher Scientific, Waltham, MA, USA). The presence of a single sharp peak in the melt curve was used to confirm primer specificity. Three biological replicates were analyzed for each experiment and three technical replicates for each biological replicate. Calculation of the relative expression level of the target gene was based on the 2^–ΔΔCt^ method (Livak and Schmittgen, 2001).

### RNA interference and bioassays

According to the DEGs analysis, a host gene that was significantly upregulated after fungal infection was chose for further function analysis by RNAi-mediated gene knockdown. The double-strand RNA (dsRNA) of a serine protease encoding gene *NlSPN* with the length of 495 bp was synthesized using a MEGAscript T7 transcription kit (Ambion, Austin, TX, USA) according to the manufacturer’s instructions. For the negative control, the dsRNA of *GFP* gene (dsGFP) with the length of 344 bp was generated using the plasmid pCAMBIA1302 as a template. The specificity of dsRNA products were checking by blasting their sequences to the whole genome of BPH, and no long stretches of nucleic identity were observed except with their specific target sequences. Specific primers containing the T7 promoter sequence for synthesizing dsRNA were listed in Supplementary Table 1. The dsRNA products were assessed by 1% agarose gel electrophoresis, quantified using a NanoDrop 2000 spectrophotometer and confirmed by sequencing.

RNAi experiments were performed by injecting 100 ng of dsRNA (20 nL with the concentration of 5,000 ng/μL) into the abdomen of each newly emerged adult (24 h after emergence) using a manual microinjector (Nanoliter 2000 Injector, WPI Inc., Sarasota, FL, USA). After dsRNA microinjection, the adults were placed on TN1 rice seedlings and reared under the conditions of 27 ± 1°C, 70 ± 10% relative humidity and a 14:10 h light/dark photoperiod in the growth chamber. Any dead individuals were removed, and the survival was recorded daily for 10 days. To assess the inhibition efficiency of the dsRNA on the expression of the target gene, ten BPH adults were sampled every 2 days during a 8-day period after each dsRNA microinjection. The methods for RNA extraction and qRT-PCR analysis were the same as those described above. For co-treatments of dsRNA microinjection and fungal infection, the newly emerged adult of BPH were microinjected with a dsRNA dose of 100 ng per adult (20 nL with the concentration of 5,000 ng/μL) and then topically sprayed with 1 ml conidia suspension (1 × 10^9^ conidia mL^–1^) of the isolated fungal pathogen or 0.02% Tween 80 as control. BPH survival rates were calculated daily for 10 days after co-treatments. Each experiment repeated three times and each replicate consisted of at least 40 BPH adults.

### Statistical analysis

Percentages of BPH nymphs or adults mortality in the bioassays was adjusted using Abbott’s formula, and corrected mortality values (%) were used (Abbott, 1925). The values of median lethal time (LT_50_) for all bioassays were estimated using non-linear regression data analysis programs implemented in software GraphPad Prism 8. The statistical model of log (agonist) vs. normalized response (variable slope) was used for the parameter generation. The survival rates of BPH adults between dsNlSPN- and dsGFP-injection groups in RNAi experiment was compared using Kaplan–Meier analysis (log-rank test) by GraphPad Prism 8. DPS software was used for statistical analysis (Tang and Zhang, 2013). All data from the repeated experiments were expressed as mean ± standard error. Prior to statistical analysis, the Shapiro–Wilk test and F test were conducted to test the normality and homogeneity of all variances, respectively. The Student’s *t*-test was used to compare the data between control and treatment groups. Differences were considered to be significant at *P* < 0.05.

## Results

### Morphological and molecular identification of the fungal pathogen

**FIGURE 1:**
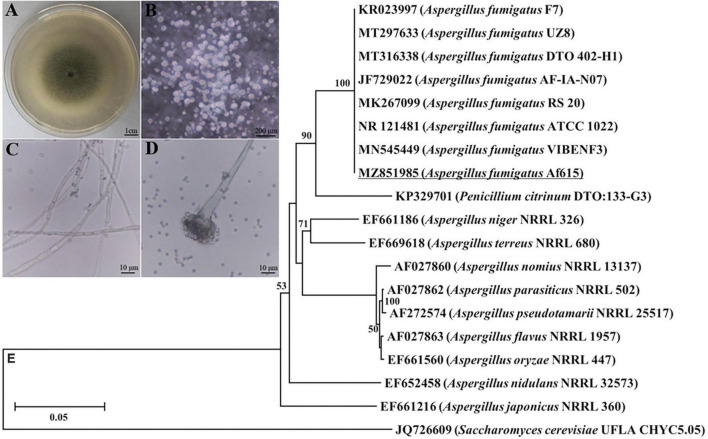
Morphological and molecular identification of the fungal pathogen form the infected cadavers of BPH. **(A)** Fungal colony grown on a PDA plate at 28°C for 7 days. **(B)** Aerial hyphae directly grew out of the PDA medium. **(C)** Hyphae. **(D)** Conidial heads and conidia. **(E)** Neighbor-joining tree based on ITS sequence of the isolated fungal strain Af615. The numbers at each branch point represents the percentage of the bootstrap values.

A pathogenic fungal strain was successfully isolated from the infected cadavers of BPH. After routine culture on PDA plates at 28°C for 5 days, the fungal colony reached an average diameter of 2.85 ± 0.15 cm and exhibited olive-green color (Figure 1A). The aerial hyphae directly grew out of the medium and the conidia grew on the phialide in chain-like conformations (Figures 1B, C). The conidia were globose or subglobose with an average diameter of 2.6 ± 0.1 μm (Figure 1D). For molecular identification, the partial ITS sequence of the fungal isolate was amplified and sequenced. The obtained sequence was 558 bp in length (GenBank accession number: MZ851985) and shared 100% similarity with those of the *Aspergillus fumigatus* strains. Phylogenetic analysis also showed that the tested fungal strain clustered with the strains of *A. fumigatus* with a bootstrap support of 100% inferred by the NJ method (Figure 1E). Combining the results of morphological and molecular characterization, the isolated fungal strain was identified as *A. fumigatus* and named as *A. fumigatus* 615 (designated Af615 herein).

### Virulence of the fungal pathogen against BPH

**FIGURE 2:**
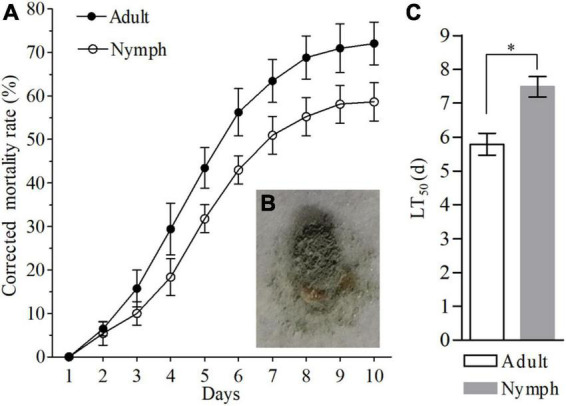
Pathogenic effects of Af615 strain on the third-instar nymphs and newly-emerged adults of BPH. **(A)** The corrected mortality rates of BPH nymphs and adults infected by Af615 during a 10-day observation period. **(B)** The symptom of BPH adult infected by Af615. **(C)** The LT_50_ of BPH nymphs and adults after Af615 infection. Error bars: SD of the mean from three replicates. The asterisk above bars indicates significant difference (*P* < 0.05, *t*-test).

The insecticide effect of Af615 against nymphs and adults of BPH were illustrated in Figure 2. The mortalities of nymphs and adults of BPH both generally increased with the post-treatment time during a 10-day observation period. Most of the BPH nymphs and adults were alive after 1 day post-infection (dpi), followed by a significant decrease at 4 dpi (Figure 2A). The corrected mortalities of 50.94 ± 4.32% for the third-instar nymphs and 63.44 ± 4.63% for newly emerged adults were observed at 7 dpi, respectively. The BPH individuals killed by Af615 showed the typical symptoms of mycosis, being well mycotized after 5 days of incubation at saturated humidity (Figure 2B). As a result of the modeling analysis, the LT_50_ estimate of the fungal strain against nymphs was 7.49 ± 0.53 days, which was significantly higher than that against adults (5.79 ± 0.56 days) (df = 4, *t* = 3.51, *P* < 0.05, *t*-test) (Supplementary Table 2 and Figure 2C).

### General characteristics of the transcriptomic sequencing data

To obtain a thorough understanding of the transcriptomic response of BPH to fungal infection, RNA sequencing of surviving BPH adults from the control and the fungal treatments at 1, 2, 3 and 4 dpi were performed, respectively. As a result, a total of 1.08 billion raw reads were yielded from all the samples. After quality filtering, an average of 42.71 million clean reads (40.86–45.36 million) and 43.73 million clean reads (37.26–48.55 million) with a GC content of each sample ranging from 45.50 to 47.00% and a Q30 value varying from 98.16 to 98.57% were obtained from the fungal-infected samples and uninfected controls, respectively. Of those, more than 47.0% in each sample was uniquely mapped to the BPH genome (Table 1).

**TABLE 1:** Summary of RNA-seq data for BPH treated with Af615 and untreated control^†^.

Sample	Raw reads	Clean reads	Q30	GC content	Mapped to BPH genome	
					**Mapped reads** **(%)^†^**	**Unique match** **(%)^†^**
**Fungal treatment**						
An1d_1	42384298	40276670	98.16%	47.00%	32821099 (81.49%)	19229813 (47.74%)
An1d_2	49205192	47149784	98.20%	47.00%	38545016 (81.75%)	22441616 (47.60%)
An1d_3	50957614	48642322	98.16%	47.00%	39554158 (81.32%)	23221916 (47.74%)
An2d_1	47866644	45587782	98.20%	47.00%	37503265 (82.27%)	21288110 (46.70%)
An2d_2	40498850	38405888	98.32%	47.00%	31624063 (82.34%)	18198336 (47.38%)
An2d_3	41006688	38949888	98.25%	47.00%	32120364 (82.47%)	18428670 (47.31%)
An3d_1	40095844	38311204	98.25%	47.00%	31590755 (82.46%)	17848732 (46.59%)
An3d_2	48656620	46470634	98.21%	47.00%	38351231 (82.53%)	21566248 (46.41%)
An3d_3	39660794	37796814	98.17%	47.00%	31111685 (82.31%)	17468210 (46.22%)
An4d_1	51304478	48955336	98.29%	47.00%	40643625 (83.02%)	23045410 (47.07%)
An4d_2	44454198	42230404	98.26%	47.00%	35080131 (83.07%)	19855611 (47.02%)
An4d_3	42051570	39744096	98.19%	47.00%	32932687 (82.86%)	18717161 (47.09%)
**Control**						
CK1d_1	43021750	41050230	98.19%	46.00%	32807243 (79.92%)	20004912 (48.73%)
CK1d_2	34595826	33039756	98.20%	47.00%	26320036 (79.66%)	15980628 (48.37%)
CK1d_3	39540902	37689070	98.18%	47.00%	29978050 (79.54%)	18199376 (48.29%)
CK2d_1	52579468	51725552	98.50%	45.50%	42702138 (82.56%)	24085213 (46.56%)
CK2d_2	53267922	52282898	98.43%	46.00%	43293772 (82.81%)	24248128 (46.38%)
CK2d_3	42553040	41656444	98.49%	46.50%	34356732 (82.48%)	19201729 (46.10%)
CK3d_1	49074488	48070302	98.51%	46.00%	39990146 (83.19%)	22338480 (46.47%)
CK3d_2	47534432	46638950	98.53%	46.00%	38827821 (83.25%)	21515515 (46.13%)
CK3d_3	38274768	37568398	98.48%	46.00%	31231786 (83.13%)	17384245 (46.27%)
CK4d_1	40856940	40148582	98.57%	46.00%	33116755 (82.49%)	18821918 (46.88%)
CK4d_2	52456050	51436050	98.50%	45.50%	42435190 (82.50%)	24117796 (46.89%)
CK4d_3	44050612	43398306	98.46%	45.00%	35549826 (81.92%)	20134786 (46.40%)

^†^The percentage (%) for the mapped reads and the unique match is based on the count of total clean reads.

### DEGs analysis and qRT-PCR validation

**FIGURE 3:**
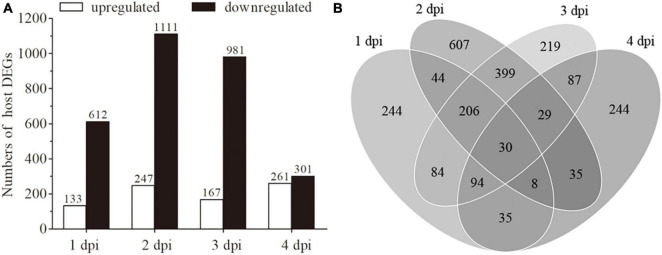
Analysis of differentially expressed genes (DEGs). **(A)** An overview of DEGs in the libraries of 1 dpi (An1d vs. CK1d), 2 dpi (An2d vs. CK2d), 3 dpi (An3d vs. CK3d) and 4 dpi (An4d vs. CK4d). **(B)** Venn diagram for the total counts of DEGs at 1, 2, 3 and 4 dpi.

To identify DEGs, the transcriptomes of fungal-infected and uninfected samples were compared at each time point (An1d vs. CK1d, An2d vs. CK2d, An3d vs. CK3d and An4d vs. CK4d) based on the standard of | log_2_ (Fold change)| > 1.0 and the adjusted *P*-value < 0.05. As shown in Figure 3A, 745 (up-/down-regulated: 133/612), 1,358 (up-/down-regulated: 247/1111), 1,148 (up-/down-regulated: 167/981) and 562 (up-/down-regulated: 261/301) genes were differentially expressed in An1d, An2d, An3d, and An4d when compared with the corresponding controls (Supplementary Table 2). Of those, 30 DEGs were consistently expressed at the four time points (Figure 3B). To validate the DEGs data, ten candidate genes selected at each time point after fungal or control treatments were quantified for their transcript levels by qRT-PCR with the primer pairs listed in Supplementary Table 1. As expected, all genes shown similar expression tendency between qRT-PCR and RNA-seq data (Supplementary Figure 1).

### Functional enrichment analysis of DEGs

Gene ontology (GO) enrichment analysis was performed to determine the potential biological functions of the DEGs in BPH after fungal challenge. Compared with the corresponding controls, the detected DEGs were assigned into 180, 232, 136 and 123 GO terms within three main categories (biological process, molecular function and cellular component) at 1, 2, 3 and 4 dpi, respectively. Within the category of biological process, the functional terms of “response to oxidative stress,” “oxidation-reduction process,” and “DNA-templated transcription, initiation” were overrepresented, while the GO terms including “DNA binding,” “ATP binding,” and “GTP binding” belonging to the category of molecular function and the GO terms of “cytoplasm,” “nucleolus,” and “cellular_component” affiliated to the category of cellular component were mainly enriched (Supplementary Table 3). KEGG enrichment analysis revealed that all the DEGs at the four post-infection time points were mapped to 14, 13, 15 and 11 pathways, respectively. Of those, the pathways involved in host metabolism and defense were significantly enriched, such as “phagosome,” “carbon metabolism,” and “insulin signaling pathway” at 1, 2, and 3 dpi, and “biosynthesis of amino acids” and “fatty acid metabolism” at 4 dpi (Supplementary Table 4).

### DEGs involved in host defense against fungal infection

To further explore the host defense mechanisms against Af615 infection, DEGs involved in host immunity and defense were analyzed. Based on comparative transcriptomic analysis, numerous genes that participated in host innate immunity, oxidative stress response, ion/molecule transportation and biological degradation and detoxification were found to be upregulated at each post-infection time point (Table 2). For instance, several immune-related genes were obviously activated in the course of fungal infection, including genes encoding serine protease (LOC111048626, LOC111059247, LOC111045766, and LOC111045189), defensin (LOC111045303), nuclear factor NF-kB (LOC111057927) and lysosome membrane protein (LOC111044179). Remarkably, the serine protease gene (LOC111048626) was consistently activated after fungal infection. The values of log_2_ (Fold change) were 3.46 at 1 dpi, 14.20 at 2 dpi, 2.80 at 3 dpi, and 3.06 at 4 dpi, respectively. Some host genes participated in oxidative stress response were also upregulated. The expressions of a superoxide dismutase (SOD) gene (LOC111064010) and a peroxidase (POD) gene (LOC111049592) were found to be significantly increased at the later stage of infection (3 and 4 dpi), and a dual oxidase (Duxo) gene (LOC111058713) was upregulated by 2.28- and 2.51-fold at 1 and 2 dpi, respectively. Additionally, genes predicted to be involved in ion/molecule transportation were observed significantly activated during the course of infection, such as a V-type proton ATPase [LOC111063454, log_2_ (Fold change) = 1.03 at 1 dpi], an amino acid transporter [LOC111061862, log_2_ (Fold change) = 1.48 at 2 dpi], a chloride channel protein [LOC111057220, log_2_ (Fold change) = 1.36 at 3 dpi] and a sodium channel protein [LOC111054483, log_2_ (Fold change) = 2.47 at 4 dpi]. Moreover, many upregulated DEGs with well-functional annotations were associated with the physiological process of degradation and detoxification. For instance, the transcript levels of an E3 ubiquitin-protein ligase gene (LOC111051992) was upregulated by 3.97-fold at 1 dpi, a perilipin (LOC111045917) was enhanced by 2.11-fold at 2 dpi, a endochitinase gene (LOC111063805) was increased by 2.95-fold at 3 dpi and three cytochrome P450 (CYP) genes (LOC111052084, LOC111052016, and LOC111055719) were activated by above 2.00-fold at 4 dpi, respectively.

**TABLE 2:** Genes involved in innate immunity, oxidative stress response, ion/molecule transportation, biological degradation detoxification in BPH response to fungal infection at 1, 2, 3 and 4 dpi, respectively^a^.

Gene ID^a^	Description	log_2_ Fold change^b^			
		**1 dpi**	**2 dpi**	**3 dpi**	**4 dpi**
**Involved in innate immunity**					
LOC111048626	Serine protease nudel	3.46	14.20	2.80	3.06
LOC111045189	Serine proteinase stubble	1.07	1.81	1.11	NS
LOC111059247	Serine protease	NS	1.05	1.28	1.12
LOC111045766	Serine protease easter	NS	1.69	1.88	1.04
LOC111060578	Späetzle 3	NS	NS	2.10	1.14
LOC111044297	Nitric oxide synthase	NS	1.22	NS	NS
LOC111057927	Nuclear factor NF-kappa-B p100 subunit	1.26	1.39	NS	NS
LOC111044179	Lysosome membrane protein 2	NS	1.28	1.26	NS
LOC111050297	Lysosomal alpha-mannosidase	NS	NS	1.24	1.26
LOC111045303	Defensin	NS	1.02	NS	−1.23
LOC111051604	Gramicidin S synthase 1	NS	1.07	NS	NS
LOC111059878	Gramicidin S synthase 2	2.49	3.15	NS	NS
LOC111051065	Serine/threonine-protein kinase	NS	NS	1.18	1.83
**Involved in oxidative stress response**					
LOC111058713	Dual oxidase	1.19	1.33	NS	NS
LOC111064010	Superoxide dismutase	1.11	NS	1.91	1.87
LOC111049592	Chorion peroxidase	NS	1.27	NS	1.06
**Involved in ion/molecule transportation**					
LOC111063454	V-type proton ATPase	1.03	NS	1.58	1.69
LOC111061862	Cationic amino acid transporter 2	1.40	1.48	1.80	1.47
LOC111054261	Major facilitator superfamily protein	1.00	1.04	NS	NS
LOC111057220	Chloride channel CLIC-like protein 1	NS	NS	1.36	1.21
LOC111064043	Sodium- and chloride-dependent transporter	NS	NS	1.12	1.73
LOC111054483	Sodium channel protein Nach-like	NS	NS	1.08	2.74
LOC111061502	Major facilitator superfamily protein	1.64	NS	1.60	1.33
LOC111057094	Major facilitator superfamily protein	1.23	1.54	1.60	1.04
LOC111044220	Multidrug resistance protein 1	NS	1.10	1.18	1.04
**Involved in biological degradation and detoxification**					
LOC111051992	E3 ubiquitin-protein ligase HUWE1	1.99	1.06	1.57	1.14
LOC111054206	Ubiquitin carboxyl-terminal hydrolase 19	1.42	NS	1.53	1.14
LOC111058528	E3 ubiquitin-protein ligase HUWE1	1.28	1.34	1.03	1.17
LOC111063805	Endochitinase A	NS	1.27	1.56	1.01
LOC111045917	Perilipin-4	1.34	1.08	2.07	2.50
LOC111052084	Cytochrome P450 4C1-like	NS	−2.51	1.30	1.80
LOC111052016	Cytochrome P450 18a1	NS	1.77	1.27	1.24
LOC111055719	Cytochrome P450 4c3	NS	NS	1.01	1.08

^a^DEGs with the expressions up-/down-regulated over two-fold (|log_2_ Fold change| > 1.0) at least at one time point after fungal infection were shown.

^b^NS means not significant.

### Candidate gene silencing increases BPH susceptibility to fungal infection

**FIGURE 4:**
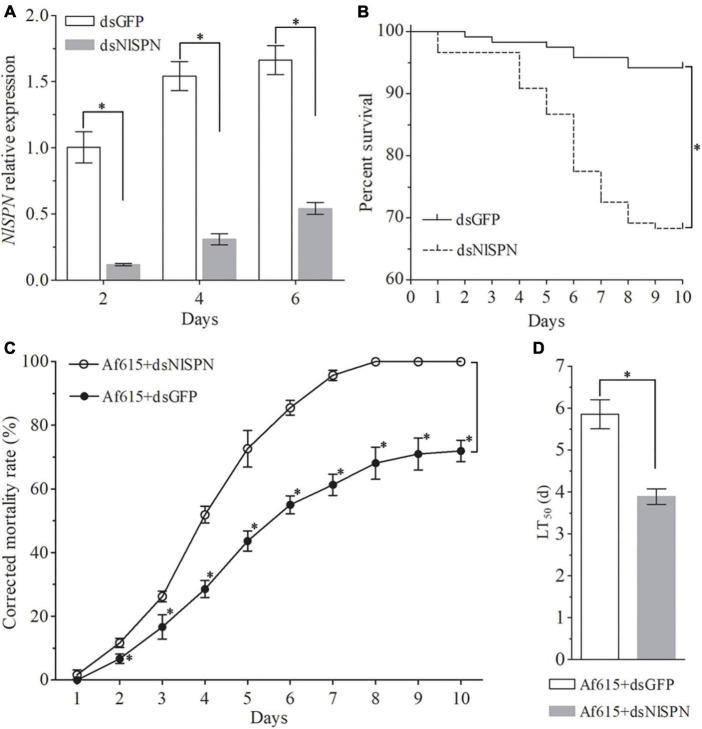
Effect of silencing a serine protease nudel gene *NlSPN* on BPH survival and susceptibility to fungal infection. **(A)** Relative expression levels of *NlSPN* at different time points after dsRNA injection. **(B)** The survival rates of BPH adults during a 10-day period of dsRNA injection. **(C)** The corrected mortality rates and **(D)** LT_50_ values (day) of BPH adults during a 10-day period of co-treated with dsRNA injection and fungal infection. Error bars: SD from three repeated assays. The asterisk above bars indicates significant difference between each group or each time point (*P* < 0.05, *t*-test).

To further explore the molecular mechanism of BPH response to fungal infection, a serine protease nudel gene *NlSPN* (LOC111048626) that was significantly upregulated at all the four post-infection time points was chose for further function analysis by RNAi. The inhibition efficiency of *NlSPN* by RNAi was determined by qRT-PCR. The results showed that the expression level of *NlSPN* was extremely decreased by 88.25% at 2 days after dsNlSPN injection when compared with dsGFP injection. Although the inhibition efficiency of *NlSPN* tended to weakened with the extension of post-treatment time, an approximate 80% decrease and more than 65% reduction in target transcript was achieved at 4 and 6 days post-treatment, respectively (Figure 4A). Meanwhile, dsRNA-mediated knockdown of *NlSPN* caused a significant decrease in survival rate of BPH adults (χ^2^ = 26.20, df = 1, *P* < 0.05) (Figure 4B). At 10 days post-treatment, the survival rate of BPH adults in dsNlSPN-injection treatment was 68.33 ± 6.29%, which was significantly lower than that of control adults treated with dsGFP (94.17 ± 1.44%) (df = 4, *t* = 6.93, *P* < 0.05, *t*-test). Laboratory bioassays showed that the final corrected mortality rate of BPH at 6 days post-treatment was 85.49 ± 1.36% for dsNlSPN + Af615 co-treatment, which was significantly higher than that for dsRNA injection (13.51 ± 5.41%) alone (df = 4, *t* = −22.36, *P* < 0.05, *t*-test) or fungal infection (55.00 ± 2.81%) alone (df = 4, *t* = −16.89, *P* < 0.05, *t*-test) (Figure 4C). Probit analysis revealed the LT_50_ value of 3.89 ± 0.18 days for dsNlSPN + Af615 co-treatment was 33.53% shorter than the estimate from dsGFP + Af615 treatment (5.85 ± 0.35 days) (df = 4, *t* = 8.64, *P* < 0.05, *t*-test) (Supplementary Table 6 and Figure 4D).

## Discussion

Nowadays, with the rapid development of insecticide resistance, biocontrol agents as an alternative to chemical pesticide against BPH are urgently needed. In the present study, we isolated and identified a native fungal pathogen from infected BPH cadavers, followed by the evaluation of pathogenicity against BPH at both nymphal and adult stages. To further explore the insect-fungus interaction, comparative transcriptomic analysis by RNA-seq was performed to determine the gene expression profiling of BPH adults response to fungal infection, resulting in a long list of DEGs which might be serve as potential gene targets for BPH biocontrol.

Based on morphological characteristics and molecular analysis, the isolated fungus was identified as a strain of *A. fumigatus* (Af615). To date, a diverse group of insects has been reported to be successfully infected by fungal entomopathogens belonging to the genus of *Aspergillus*. For instance, *Aspergillus oryzae* originally isolated from dead insect bodies exhibited high insecticidal activity toward the poultry red mite *Dermanyssus gallinae* and the migratory locust *Locusta migratoria* (Zhang P. et al., 2015; Wang et al., 2019), while *Aspergillus flavus* displayed high virulence to the silkworm *Bombyx mori* and some coconut pests (Kulshrestha and Pathak, 1997; Gupta and Gopal, 2002). To the best of our knowledge, the strain Af615 was the first record as a native fungal pathogen against BPH. The laboratory bioassays confirmed that the isolated strain Af615 displayed a high insecticidal effect toward both BPH nymphs and adults, therefore being of a potential use for biocontrol of this rice pest. However, the BPH adults showed a significantly higher susceptibility than nymphs to Af615-infection. The phenomenon of the increased mortality of host insect in the adult stage compared to the nymphal stage was also observed in other insect-fungus systems (Immediato et al., 2015; Wang et al., 2019). The shift of fungal virulence according to the host development stage could be explained on different aspects. One important reason might be the present of the molting process at the nymphal stage that could reduce the adhesion of conidia to the body surface.

The laboratory bioassays conducted here were also used to determine the time points of sampling for transcriptomic sequencing. The mortality trend showed that almost all of the BPH adults were alive at 1 dpi, followed by a significant decrease at 4 dpi, suggesting that 1–4 dpi could be critical time period for the interaction between BPH and Af615. Hence, the time points of 1 and 2 dpi were chose as representative of the initiation of infection, 4 dpi were sampling as the time point representing the intense interaction between insect and fungus, and 3 dpi was also selected for considering the earlier response of BPH to fungal infection. The amount and high mapped ratios of the transcriptomic sequencing data suggested that the RNA-seq data obtained in the present study was abundant and credible. The qRT-PCR validation results further confirmed that the sequencing data was reliable for further analysis of the host-pathogen interaction. Subsequently, we identified numerous host genes that shown significant alterations in expression during the BPH response to Af615-infection. Of those, DEGs related to host immunity and defense with a high fold change warrant to be further and extensively investigated.

In the long-term evolutionary process, insects have developed a safe and effective innate immune system to defense pathogens and adverse conditions (Sheehan et al., 2020). Of those, the prophenoloxidase (PPO) cascade activation and Toll pathway initiation are two critical insect immune responses against the invading pathogens, which are mediated by serine proteinase cascades (Cao et al., 2017). In addition to being important components of humoral immunity, serine protease also participated in cellular immunity like phagocytosis (Tokunaga et al., 2021). Based on comparative transcriptomic analysis, we found that the mRNA abundances of four serine protease genes in BPH were significantly upregulated in the course of fungal infection and one of them (LOC111048626) was consistently activated at all point times post-infection. This finding highly suggested that serine protease cascade-mediated activation of innate immunity in BPH plays crucial roles in host defense against the Af615-attack. Additionally, microbial infection could induce the production of anti-microbial proteins (AMPs) or other defense molecules in insect hemocytes and fat body through the Toll and IMD pathways by the activation of NF-kB transcription factors (Wu et al., 2018). For instance, AMPs such as gloverin, moricin, lebocin and attacin in the silkworm were significantly induced by both *Bacillus thuringiensis* and *Pseudomonas aeruginosa* (Nesa et al., 2020). In this study, the abundance of a defensin gene (LOC111045303) was found to be associated with the time post-infection. Its expression was significantly activated at the earlier stage of fungal infection (2 dpi), as well as a NF-kB gene (LOC111057927). However, with the extension of the post-infection time, the up-regulation effect of gene expression disappeared. Similar mode of action of this defensin gene was also observed in BPH response to another fungal entomopathogen *M. anisopliae* (Wang Z. L. et al., 2021). Undoubtedly, future works focusing on the precise biological functions of these immune-related genes are urgently needed.

The generation of reactive oxygen species (ROS) in insects is another defense strategy to combat microbial infection (Butt et al., 2016). Hydrogen peroxide (H_2_O_2_), a main source of ROS, was mainly produced by dual oxidase (Duxo) in the insect body cavity. The silkworm demonstrated an upregulated *Duox* expression and raised H_2_O_2_ concentration by bacterial challenge (Zhang L. et al., 2015). Silencing *Duxo* gene in the diamondback moth *Plutella xylostella* could significantly increase host susceptibility to *B. thuringiensis* infection (Sajjadian and Kim, 2020). In this study, a *Duxo* gene (LOC111058713) in BPH was found to be significantly upregulated at 1 and 2 dpi, implying that Duox-derived ROS play an important role in BPH defense against Af615 at the earlier stage of infection. Generally, ROS in insects at low concentration levels can efficiently kill invading pathogens, yet excessive levels are harmful to the host itself by disruption of biomacromolecule structure and microbial homeostasis (Li et al., 2020). The increased in the expression levels of antioxidant enzymes such as genes encoding SOD (LOC111064010) and POD (LOC111049592) detected in the present study might be responsible for regulating biologic redox equilibrium and preventing against oxidant injuries during the process of fungal pathogen defense.

Many DEGs known to be involved in molecule transportation and xenobiotics detoxification were also detected in BPH-Af615 interaction. For instance, genes encoding transporter-related proteins, including major facilitator superfamily proteins (LOC111061502, LOC111054261, and LOC111057094) and multidrug resistance protein (LOC111044220) in BPH increased their expressions significantly after fungal infection. As is well known, membrane transporters can facilitate the diffusion of specific molecules across cellular membranes, and are implicated in host resistance to pathological and other adverse conditions (Hu and Xia, 2019). For instance, five genes encoding transporters or permease-related proteins in the ghost moth *Thitarodes* larvae were significantly enhanced their expressions upon the infection by a fungal entomopathogen *Ophiocordyceps sinensis* (Zhong et al., 2016). Insect CYPs are multi-function enzymes involved in insect growth, development, pathogenesis and environmental adaptation, especially in metabolic detoxification of xenobiotics (Lu et al., 2021). In our study, the expression levels of three CYP genes (LOC111052084, LOC111052016, and LOC111055719) were also found to be significantly upregulated in Af615-infected BPH when compared to the control. Similar activation patterns of CYP expression were also observed in other insect-fungus interactions. For example, the expression levels of CYPs were highly induced in the diamondback moth and bamboo wireworm in response to fungal infection (Chu et al., 2016; Ye et al., 2018). Hence, our results highlighted a significant of these transportation and detoxification related genes for the host defense against the fungal pathogen in BPH.

RNAi-mediated pest control has been proven to be an effective and eco-friendly method for pest control due to its target specificity and easy degradation (Mamta and Rajam, 2017). Generally, the efficacy of RNAi-based strategy depends on the target gene selection and method of dsRNAs delivery (Liu et al., 2020). Undoubtedly, our transcriptomic data provided a valuable genetic resource of putative targets that might be exploited *via* RNAi. A dramatic decrease in BPH survival rate after gene silence of *NlSPN* highly indicated that *NlSPN* could be used as a potential RNAi target for biocontrolling of BPH. Direct application of dsRNA and expression of dsRNA in transgenic organism were proved to be feasible in both field and laboratory settings to control insects (Adeyinka et al., 2020). Recent studies revealed that recombinant fungal entomopathogen expressing dsRNA was an ideal choice for dsRNA delivery due to additive effects of fungal infection and RNAi (Hu and Wu, 2016). For example, recombinant *Isaria fumosorosea* strain expressing dsRNA targeting to a Toll-like receptor gene TLR7 in *Bemisia tabaci* could significantly enhanced the whiteflies mortality (Chen et al., 2015). The additive effect of RNAi and fungal infection was also observed in the present study, as higher mortality was found when the BPH adults co-treated with ds*NlSPN* injection and Af615 infection. Construction and bioassay of recombinant strains of Af615 expressing a specific BPH dsRNA warranted to be investigated in further study.

## Conclusion

In the present study, we isolated a native fungal pathogen against BPH and identified as *A. fumigatus*. The isolated strain was highly infective to BPH both at nymphal and adult stages under laboratory conditions and appeared to be of potential use for BPH biocontrol. Transcriptomic analyses provided insight into a genome-wide interaction between the BPH and *A. fumigatus* and found a large number of DEGs that mainly involved in host immune defense and cell detoxification. Suppression of an upregulated DEG (*NlSPN*) encoding a serine protease could result in a significant decrease in BPH survival. The additive effect of RNAi and fungal infection provided new clues to develop pest management strategy against BPH.

## Data availability statement

The datasets presented in this study can be found in online repositories. The names of the repository/repositories and accession number(s) can be found in the article/Supplementary material.

## Author contributions

Z-LW, G-FL, and X-PY conceived and designed the experiments. Z-LW, Y-DW, Y-QC, and Z-HY performed the experiments and analyzed the data. Z-LW wrote the manuscript. G-FL and X-PY reviewed and edited the manuscript. All authors read and agreed to the published version of the manuscript.

## Footnotes

1
http://blast.ncbi.nlm.nih.gov/blast.cgi

2
http://www.megasoftware.net

## Supplementary material

Supplementary Figure 1 Verification of 10 randomly selected DEGs by qRT-PCR and RNA-seq data. All selected DEGs are detailed in Supplementary Table 1.

Supplementary Table 3 The DEGs from BPH infected by Af615 at 1, 2, 3, and 4 days post infection, respectively.

Supplementary Table 4 GO analysis of DEGs from BPH infected by Af615 at 1, 2, 3, and 4 days post infection, respectively.

Supplementary Table 5 KEGG pathway enrichment analysis of DEGs from BPH infected by Af615 at 1, 2, 3, and 4 days post infection, respectively.
