# Supplementary material for: Characterization and transcriptomic analysis of a native fungal pathogen against the rice pest Nilaparvata lugens
Source: Front Microbiol. 2023 May 18;14:1162113. doi: 10.3389/fmicb.2023.1162113 (PMC10232905; doi:10.3389/fmicb.2023.1162113)
Supplement: Supplementary file 1 [file Table_1.doc]

**Table S1.** Specific primer pairs used in this study.

| **Gene ID** | **Description** | **Sense/Antisense primer sequences (5′-3′)** | **Product length (bp)** | **Purpose** |
| --- | --- | --- | --- | --- |
| LOC111048626 | serine protease nudel | ACAGACCGGAGATTTATCAGTCG / AGATGCAGGAGGTTTTTCAGGT | 190 | qRT-PCR validation |
| LOC111056357 | titin | GAGTCAACCAGTAACCCAATC / TTCTGAACAGTGAACCCATCT | 123 |
| LOC111051992 | E3 ubiquitin-protein ligase HUWE1 | CATTCTGCGTCTGAAACCAAT / AACAGTCTGCCAAACCAAAGC | 107 |
| LOC111055973 | glutenin | GCCATCCACCAACAACATTAT / TTGTGGTCGGTGAATAGACAT | 149 |
| LOC111052170 | cubilin | CGGAGATTATGTTCGGGTATT / CTGGCAGTTCGATGTTTAGTG | 147 |
| LOC111055219 | uricase | CAGTTTGCGTTGATTCTGTGC / ATAAATGCGTGATTGTGAGGA | 131 |
| LOC111058170 | pupal cuticle protein 36a | CAGCCAAACCTTGGAGATGGA / GTCGGTGCCCGTGTAGGAGAA | 144 |
| LOC111049567 | dual specificity protein phosphatase | GATGGCTGCCTGCTACCTGAT / CTTCTCCTGGTCGTAGGTCTCG | 109 |
| LOC111050948 | interleukin-3-regulated protein | GCGAAACTATGCTATGGAGATGG / GGCGTTGGGTGTTGAACTGGA | 107 |
| LOC111054905 | flightin | TATCTGCCGACACTGGGTTAG / CAGTCCTTTGTTGCGTTTATC | 115 |
| Reference gene | 18S ribosomal protein | GTAACCCGCTGAACCTCC / GTCCGAAGACCTCACTAAATCA | 170 |
| LOC111048626 | serine protease nudel | GGATCCTAATACGACTCACTATAGGGTGGAAGACGTTGTCAATGAG / GGATCCTAATACGACTCACTATAGGGCAGGAGGTTTTTCAGGTTT | 495 | dsRNA synthesis |
| Control gene | green fluorescent protein gene | GGATCCTAATACGACTCACTATAGGGATACGTGCAGGAGAGGAC / GGATCCTAATACGACTCACTATAGGGCAGATTGTGTGGACAGG | 344 |
