# Supplementary material for: Characterization and transcriptomic analysis of a native fungal pathogen against the rice pest Nilaparvata lugens
Source: Front Microbiol. 2023 May 18;14:1162113. doi: 10.3389/fmicb.2023.1162113 (PMC10232905; doi:10.3389/fmicb.2023.1162113)
Supplement: Supplementary file 2 [file Table_2.doc]

**Table S2**. Medium lethal time (LT_50_) values for the fungal pathogen Af615 against BPH nymphs and adults.

| **Sample** | **LT_50_ (day)** | **Mean LT_50_ (day)** † | **95% confidence limit** | |
| --- | --- | --- | --- | --- |
|  |  |  | **Lower** | **Upper** |
| Nymph | 8.08 | 7.49±0.53^a^ | 7.38 | 8.85 |
|  | 7.29 |  | 6.72 | 7.91 |
|  | 7.09 |  | 6.67 | 7.54 |
| Adult | 6.35 | 5.79±0.56^b^ | 6.01 | 6.72 |
|  | 5.78 |  | 5.36 | 6.23 |
|  | 5.23 |  | 5.04 | 5.43 |

† Mean±SD was estimated from three replicate bioassays. Means with different letters in the same column indicate significant difference (df=4, t=3.51, *P*<0.05, *t*- test).
