# Supplementary material for: Characterization and transcriptomic analysis of a native fungal pathogen against the rice pest Nilaparvata lugens
Source: Front Microbiol. 2023 May 18;14:1162113. doi: 10.3389/fmicb.2023.1162113 (PMC10232905; doi:10.3389/fmicb.2023.1162113)
Supplement: Supplementary file 3 [file Table_6.doc]

**Table S6**. Medium lethal time (LT_50_) values for BPH adults co-treated with dsRNA injection and fungal infection.

| **Sample** | **LT_50_ (day)** | **Mean LT_50_ (day)** † | **95% confidence limit** | |
| --- | --- | --- | --- | --- |
|  |  |  | **Lower** | **Upper** |
| Af615+dsGFP | 6.26 | 5.85± 0.35^a^ | 5.92 | 6.61 |
|  | 5.62 |  | 5.29 | 5.97 |
|  | 5.69 |  | 5.44 | 5.95 |
| Af615+dsNlSPN | 3.78 | 3.89±0.18^b^ | 3.59 | 3.98 |
|  | 4.10 |  | 3.81 | 4.23 |
|  | 3.79 |  | 3.62 | 3.97 |

† Mean±SD was estimated from three replicate bioassays. Means with different letters in the same column indicate significant difference (df=4, t=8.64, *P*<0.05, *t*- test).
