# Supplementary figures and images for: Characterization and transcriptomic analysis of a native fungal pathogen against the rice pest Nilaparvata lugens
Source: Front Microbiol. 2023 May 18;14:1162113. doi: 10.3389/fmicb.2023.1162113 (PMC10232905; doi:10.3389/fmicb.2023.1162113)

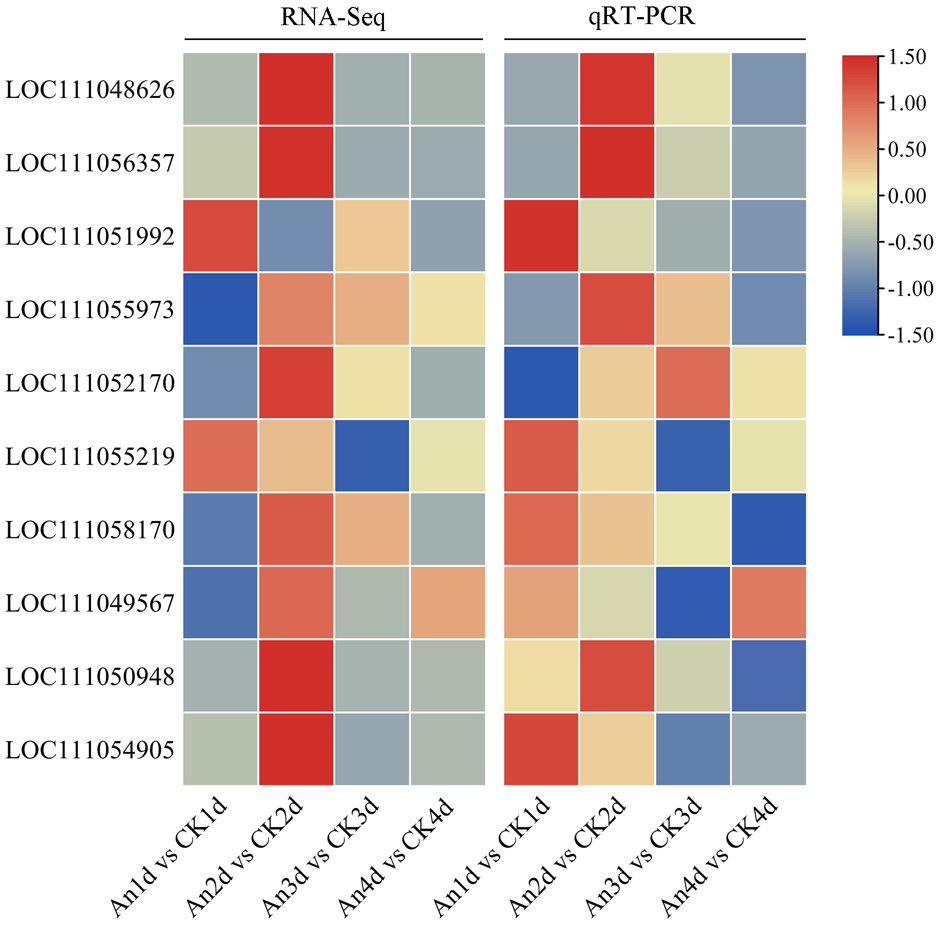

Supplement: Supplementary Figure 1 — Verification of 10 randomly selected DEGs by qRT-PCR and RNA-seq data. All selected DEGs are detailed in Supplementary Table 1. [file Image_1.TIF]
